# I Am a Mother Because I Wanted to—I Am a Grandmother Because Others See Me That Way—Motherhood as a Critical Life Event for Ageing Women

**DOI:** 10.3390/ijerph192416381

**Published:** 2022-12-07

**Authors:** Aleksandra Błachnio, Karolina Kuryś-Szyncel

**Affiliations:** 1 Faculty of Psychology, Kazimierz Wielki University, 85-064 Bydgoszcz, Poland; 2 Faculty of Educational Studies, Adam Mickiewicz University, 61-712 Poznań, Poland

**Keywords:** ageing, biographical research, critical life event, older women, intergenerational research, mothering, narrative, qualitative

## Abstract

The purpose of this study was to analyse women’s perceptions of their transition to motherhood in the late decades of their female adulthood. The research problem was whether and to what extent the meaning of the transition to motherhood changes for women from different birth cohorts. Our sample included mothers from two generational cohorts: 50 women in middle adulthood (M = 47.66), and 52 women in late adulthood (M = 69.35). The results were analysed using qualitative data analysis methods, including frequency analysis and qualitative narrative analysis. Based on the analyses, the category of transition to motherhood was found to be a standard feature for all the women studied in middle and late adulthood. In both older generations, some women recalled the birth of their first child as a coping with change. Indicators of criticality were extracted from their narratives: potential bivalence, the unpredictability of consequences, and longevity of life changes. The results showed that the generation of women in late adulthood exhibited significantly fewer difficulties related to their own motherhood. The semantic dominant of the narratives studied is motherhood as taking responsibility for another person.

## 1. Introduction

The biographical transition to motherhood is defined as the transition from a woman-non-mother to a woman-mother; it is indicated by the moment she has her first child. It has been fundamental for the personal and social development of women throughout their lives. The ongoing civilization changes have been transforming the fields in which women have been functioning socially and professionally and deconstructing their developmental tasks, making motherhood one of the possible biographical solutions. Women become mature in a global society where families are mostly nuclear, and where individuals prefer work-based autonomy [1]. Experiencing motherhood is no longer a normative component of life and women without children are no longer perceived as being inherently “less” or lacking [2]. Instead, it has become a potential and choice-based life scenario. A woman may want to become a mother; she may postpone this possibility for the future, or, by choosing a “childfree” scenario [3], she may entirely abandon this facet of her life. At the same time, the pluralization of life paths undermines the normativity of what was not long ago described as the “proper” social, economic, material, sexual, etc., circumstances of motherhood [4,5,6].

Research conducted currently both in Poland and in the world has emphasized the weight (salience) of this event, indicating many changes, including those related to personality, which occur in women’s lives following childbirth [7,8,9,10]. Motherhood has been described as a turning point in a woman’s life [11]. It was defined as a significant experience and a biographical life event [12,13,14], and more and more attention has been paid to the self-creative aspect of motherhood [15,16]. The research mainly focuses on the women’s “rightful” role as mothers between the ages of 20 and 35, which is emphasized as the “optimum age” for childbearing [17,18,19,20,21]. Much less attention is given to women who choose to become mothers after the age of 35 [22,23,24,25,26]. Older mothers are women who have put off motherhood until they complete their educational path and advance in their careers. The role of the mother is crucial in the upbringing of the youngest and most dependent children, and is critical as the offspring mature and enter the formative stages of their identity [27]. Later, it loses focus and becomes increasingly invisible to others. Late-life stages in womanhood include a diminution of mothering due to children growing up and they are leaving the family home [28]. The importance of adult children is only investigated in relation to the well-being, emotional and social loneliness, and mental health of older adults [29].

Discussing the experience of motherhood is narrowed down to describing the transition from a non-mother to a mother. However, there is a lack of research and analysis on the experience of motherhood from a biographical perspective, as a closer or further retrospective. It is therefore interesting to investigate differences in women’s perceptions of their transition to motherhood at the late stages of their female adulthood. In particular, it is worth asking about the meaning of motherhood in the subjective assessment of postmenopausal women, where social discourse has seen motherhood belittled and the role of grandmother magnified.

In late adulthood, women are mainly inscribed in the role of grandmother [30], often even ignoring the role of mother, which they carry out for many years and often even decades. Meanwhile, as emphasized by Astrid Tokaj and Danuta Krzysztofiak [31], despite the passage of time and taking on new roles, women continue to be mothers. This experience, that of “senior motherhood”, is reflective and individualized. The literature describes its different types: fulfilled and mature motherhood; intrusive (overprotective) motherhood; occasional motherhood; forgotten motherhood; protective motherhood; surrogate motherhood (grandmother in the role of mother for her grandchildren); reverse motherhood (a grandmother in the role of a mother for her adult child’s child); reverse motherhood (an old mother in the part of a child of her children) [31]. 

The studies devoted to the evolution and changes in the perception of one’s own motherhood (or rather, of oneself as a mother) in the course of life are scarce. To fill this gap in the analysis of the transition to motherhood, we employed Leoni Sugarman’s Seven-Phase Model of Stages Accompanying Transition [32]. The author emphasizes that despite the closure of the transition process through the integration of experience, the process may continue: “*The transition has become integrated into the living space and no longer dominates it. However, it is also possible to question whether transitions never end (…). This is compatible with the notion of the life course as a never-ending story and the inevitably temporary nature of life structures*” [32] (p. 148). The continuity of the process of reconstructing the meaning of this event in a woman’s biography is linked to the subjective and cultural weight that women attribute to the transition to motherhood. The research we conducted confirmed this belief. The problem that we made the subject of our research concerned the extent to which the women we studied in the subsequent decades of their adult lives (in middle and late adulthood) continuously indicate the birth of a child as a critical life event.

In this study, we assess the retrospective statements about the biographical event of the transition to motherhood itself. Therefore, we formulated a research problem concerning whether and to what extent the significance of the event, which is becoming a mother, is changing for women from different birth cohorts. We posed a research question: Is the length of the time perspective (scope of retrospection) relevant to distinguishing this event as a turning point in one’s own biography, and if so, to what extent? According to the concept by Sigrun-Heide Filipp [33], a critical life event is one that stops a person’s previous activity and requires the use of new, previously unknown coping methods since the current forms of behaviour become insufficient. Therefore, the turning point leads to changes resulting from coping and can be both progressive and regressive.

## 2. Materials and Methods

The study aimed to compare the significance of the transition to motherhood in the individual biographies of women in middle (40–59 years) as well as late (60 years and above) adulthood. The chronological frames of these periods are in accordance with the literature review [34,35]. We were interested in whether the memory of giving birth being several dozen years away chronologically changes the way its valency regarding the subjective assessments among the persons. The biographical narrative methodology involved two different readings of the interview transcript. The first reading sought to establish the overall “story” told in the interview and to consider the researcher’s reactions to it. The second reading explored how the respondent recalled and valued herself as a mother, specifically if motherhood she perceived as a critical life event. This sequential analysis of qualitative data was intended to “reveal greater complexity and depth of understanding” of the research problem [36] (p. 120). To obtain an intergenerational perspective, in our measurement, we involved mothers from two generational cohorts: 50 women in middle adulthood (M = 47.66), and 52 women in late adulthood (M = 69.35). The research sample included women who had at least one child and spoke of the birth of a child as a critical life event. The second inclusion criterion regarding the content of the narratives was necessary because women were free to choose both the time perspective and the critical life events they talked about. Data were collected using the semi-structured thematic biographical interview method [13,37]. We aimed to obtain the narratives about the life-turning events in the biographies of adults representing generations of adult daughters and ageing mothers within a given family system. The research was conducted in 2019. A demographic description of the samples is presented in Table 1.

In the intergenerational project, the researcher asked the person to indicate important, significant events in her biography and then encouraged the person to choose from among the listed events the ones she would like to share (one or more) [37]. It turned out the vast majority of women focused on their transition to motherhood. The results were analysed using qualitative data analysis methods, including frequency and categorical narrative analysis. Deductive content analysis (theory-driven) based on the criticality indicators extracted after S.H. Filipp [33], enriched with inductively generated indicators based on analysis of the statements of the women surveyed (102 samples), was used. On the basis of the theory, the following categories emerged: novelty/suddenness/unexpected event; life change, potential bivalence: personal gain (enrichment of oneself as a person); loss (loss of previous activity); the need to develop new coping strategies. On the basis of inductive analysis, a category emerged: the transformation of life orientation/perspective, i.e., from egoism to altruism, and the category of a developmental stimulator. The latter two categories can serve as a specific, hitherto unknown indicator of a woman’s transition to becoming a mother.

## 3. Results

The women participating in the study had the freedom to choose both the time perspective to which they would refer in their narratives (although they were encouraged to use the broadest possible, lifetime perspective), as well as the events they would talk about in their descriptions. In spite of this, the vast majority of them focused on their transition to motherhood.

### 3.1. Motherhood—A New, Sudden, and Unexpected Event

The first indicator of criticality according to S.H. Filipp’s theory is the suddenness of the event, which is new and unexpected to the individual [33]. Despite the fact that the birth of a child is preceded by a series of other events, and it can take nine months of pregnancy for a woman to become accustomed to the idea of her own motherhood, the women surveyed perceive the fact of becoming a mother as a sudden and unexpected event. Their feelings may be the result of novelty, but also the result of a change in habits and ways of doing things (the cessation of previous activities and the development of the mother’s strategies for coping) [11].

The narratives quoted depict such a moment of astonishment, typical of critical life events. The situation in which the surveyed women found themselves was unexpected and unplanned, and this suddenness caused their previous activities to stop and it was necessary to introduce changes in their lives. One of the speakers titles an event from the past, “Unexpected children in my life,” and completes her statement:

*I don’t know if I have succeeded it, perhaps I can’t manage it. I have no idea. It seems to me that somehow I put it in order, I synchronized all my activities (…) I owe a lot to my older sons (…) Because they help me (…) They look after the child sometimes or pick [my kid] up from the kindergarten (…) I thought I could control it all. Mostly the responsibilities. The responsibilities, work, everything (…).*

*(woman, 41 yo [11])*

### 3.2. Motherhood-like Life Changes

The second indicator of criticality found in the analysed narratives is life change. Recall that, according to S.H. Filipp [33], this change can be positive, negative, or ambivalent, and involves the potential bivalence of critical life events. In the narratives of the women surveyed, we find all the indicators mentioned.

Most of the surveyed women from the 40-plus as well as from the 60-plus generation have indicated that a life-turning event, which is giving birth to a child, means **a change for the better** for them. Women view the transition from being a woman to being a woman and mother in terms of profit. They mention enrichment, complementing, and receiving love from a child. “Better” means a life that is both fuller and richer with new, previously unknown experiences and more complete with the presence of an object of unconditional love, which, according to the person, is eternal love.

Here are some typical narratives:

*Then the birth of children (…) yes (…). It was a long time ago; they also changed… well, they changed a lot for the better, the birth of children, (…) children were adorable (…).*

*(woman, 65 yo [13])*

*Yes, especially the first child was (…) made a significant change because the whole life had to be arranged in a completely different way. you know, when a new baby appears in the family, everything in the house is subordinate to this new person. I guess you didn’t know what would happen to you, really, but it’s a wonderful time.*

*(woman, 45 yo [16])*

*Children were very important in my life. They gave me a sense of fulfillment in my life. In a word, they gave me fulfillment in my family life because there would also be many such moments in my professional career. Still, I consider family to be the most important. Joy, happiness, satisfaction, solely positive ones, and the awareness of great responsibility for a new, full family; rather positive emotions and feelings.*

*(woman, 73 yo [21])*

*…the first life event was when my children were born, and I gave birth to five of them and: each one was important to me, and something changed in my life.*

(woman, 52 yo [72])

In several statements, the transition to motherhood was ambivalent in its nature. Such views were formulated especially by those who gave birth to a child who was burdened with difficulties. These difficulties were related to the birth of a sick child or unplanned motherhood at a later age. Several depictions of such narratives are presented below. In both cases, we deal with women in middle adulthood. Their descriptions clearly indicate the difficulties associated with coping with a new, unexpected situation. The following narratives constitute excerpts from the analysed interviews. They are similar to those of younger women in their structure and content. The significance of the event that the surveyed women talk about is increased by the unpleasant sensations associated with it, thus giving birth to a child does not become a change for the better for these women solely. Nevertheless, the final balance of profits and losses is positive.

*Revolutions? This is probably when I found out that I was pregnant with W. because I already had my well-behaved children, my life put in order, peace, and she turned it all upside down (…) I had to start all over again now. (…) For a moment, it was terrible, and now it’s great. (…) Well, now I would take it as positive, but before that, it was negative. (…) you mean, has life changed? Life has changed to more chaotic.*

*(woman, 41 yo [11])*

*And a turning point was that Z. was born ill, and it turned our life upside down.”*

*(woman, 42 yo [05])*

### 3.3. Motherhood-like Change in Life Perspective

Another category that emerged from the inductive analysis is the change in life perspective. This is a category that is associated with an indicator of criticality, but in the narratives of the surveyed women from both middle and late adulthood, it found a unique realization. In the analysed women’s narratives, with high frequency, the researchers found codes that included such a formulation as “I live for the child, my affairs I put aside to take care of the child”.

Their transition to motherhood showed a change in life perspective from selfish to altruistic—I live for my child.

*Well, having a baby is a huge change (…) it is very much expected, it is a very happy, joyful event, it is probably the most important moment in a person’s life—when a baby is born. And that also totally changes the priorities overall, everything… actually, you begin to live for this child.*

*(woman, 46 yo [12])*

The change in the life perspective results in a reorientation of the woman’s own activity. The persons noticed this by explaining that all their activities aimed to satisfy the child’s needs from the time he or she appeared in their lives, providing her or him with a sense of security and stability. Despite investing most of the resources in their daughter or son, women do not contemplate it in terms of cost, a loss of part of themselves, or a so-called sacrifice.

Below, we provide some typical narratives illustrating this issue:

*…not in the sense that you are giving something up, not as a sacrifice, but you want this child to have the best possible, so everything is postponed… often you make certain decisions, you endure some awkward situations… because of this child, to provide him or her with stability, so that his small person feels happy and safe.*

*(woman, 46 yo [12])*

*Having all three children, in turn, especially the first son, changed our life completely because it changed the scale of values; the most important thing was the child, the family. The times of freedom and doing what you want are over; more responsibilities appear. Well, before the baby was born, we were married, I worked, and we could do various things; we were such free birds, with no particular duties apart from work. After giving birth to my child, however, as I said, everything changed and was re-evaluated. The most important thing was family life, and only then, and caring for a child, and only then various other kinds of pleasures.*

*(woman, 62 yo [89])*

*…my daughter, when she was born, and I started taking care of her, I got rid of selfishness about myself, because I knew then that she could only count on me, that she needed me so much, that it taught me to love properly like this. Not for something but just to love because she exists.*

(woman, 50 yo [01])

In the last narrative quoted above, we see the selflessness of maternal love. In women’s narratives in middle and late adulthood, maternal commitment resonates more clearly than in the narratives of younger women. Their closeness and concern for the child are closer to Fromm’s concept of motherly love than the less mature desire and expectation of a young mother of a young mother for her child to reciprocate her feelings, typical among women in early adulthood [13]. Many years have passed from transition to motherhood in the biographies of women in middle and late adulthood, during which their relationship with the child has changed from purely caring, one-sided, and symmetrical, when the child was entirely dependent on them (usually up to about three years of age) to a partner relationship, two-sided and complementary (when the child has entered the adult stage). Frequently, this experience is additionally reinforced by entering into a relationship with an adult child who is already a parent. It can be said that in their biographies, the persons went through various shades of love for their own child and in the life review, they return to the fundamental truth about the unconditional nature of this feeling.

The following narrative serves as an apt illustration of this point:

*Since they were born, I have faced a mother’s role, and that has changed in my life. I had never been a mother before, I was not pregnant, and I didn’t know what it was like. But now it is boundless love. **Only now do I understand what motherly love and selflessness are**. I have the impression that **it gave my life more meaning**. I guess it had such a part.*

*(woman 45 yo [96])*

The transition to motherhood is connected with a change in life perspective (from selfish to altruistic—I live for my child).

*Well, having a baby is a huge change (…) it is very much expected, it is a very happy, joyful event, it is probably the most important moment in a person’s life—when a baby is born. And that also totally changes the priorities overall, everything… actually, you begin to live for this child.*

*(woman, 46 yo [12])*

The transition to motherhood is identified with the reconstruction of the sense of meaning in one’s own life.

*My son’s birth was probably the most emotional event in my life because everything had to be adjusted for the child. Entering the adult world, teaching someone what my parents did not teach me. **You have meaning in your life**; you have something to live for. you have a son whom you would like to help get a good education and teach him what is right and what is wrong*

*(woman, 47 yo [61])*

*This is how such events were in my life. The birth of a child was a great event. It was of great importance to me. It brought many different changes in my personal and professional life. **Life took on even more meaning for me; it became multidimensional**. *

*(woman, 57 yo [24])*

We talk about the transition to motherhood in the sense of identity; when a woman changes her image of herself, becoming a mother, she transforms her attitude toward the world. She becomes responsible not only for herself but also for others. One of the narrators tells about such a transformation:

*It is definitely a life-changing event for a woman when she gives birth to a child, transforms from a little girl, a gal into a woman, a mother, then you think completely differently (…) Well, then a person is responsible not for herself, but also for someone else, not for a while, for a moment, only for a lifetime, and this is just such a turning point. Extremely weighty, very, very, very painful at first, very lovely, fantastic event, beautiful for life later on, then the woman knows that she has someone she loves and who loves her (…) it changes you, you know then work, you need to be more at home, more work and do your best!*

*(woman, 50 yo [20])*

We are dealing here with a thought-provoking example of identifying femininity with natural motherhood, which is a debating point in the discussion taking place in the field of feminist concepts between the postulate of maintaining by a woman the primary female power—the power of natural reproduction (radical and cultural feminism) and the assumption that biological motherhood is a cultural construct merely serving oppressive purposes (radical and libertarian feminism). Yet, a detailed review of these notions exceeds the scope of this article [38,39].

Transitioning to motherhood makes a woman less self-centred and selfish, a lesson she tries to teach her children as well.

*…giving birth to each of my children was an enormous event for me, which changed my life, the everyday joy of raising you, it was for me something that changed me every time too (…) these were, of course, positive and negative experiences, in general, I think that they influenced **my development as a human being** in the positive sense of the word. The appearance of my children in the world also had some (…) very positive meaning (…) well, I became better (…) somehow less selfish, if it is possible that someone would not be selfish to the end by some kind of outlining herself and one’s own person, I just want to teach my children to care about others as well, not only about themselves.*

*(woman, 50 yo [33]*)

A. Mądry [40] compares the stages of motherhood to the seasons (from spring to winter) and at the same time questions this simple analogy, pointing out that in the last phase of motherhood, unlike in nature, we are not dealing with a time of dormancy and inactivity, but with a specific change in the performance of the mother’s role: “The last phase of motherhood is usually accompanied by the assumption of parental roles by children. This event generally occurs in a woman’s late adulthood (around age 60), although it is now not uncommon for mothers to take on the role of grandmother in middle adulthood (around age 40). Thus, the last phase of the mother’s role involves a change (usually a reduction) in the tasks performed directly towards adult children and co-evolves with the assumption of care and educational duties towards the offspring of one’s own children, and sometimes even involves the performance towards them of tasks ascribed to parents” [40] (p. 17).

### 3.4. Motherhood-like Developmental Stimulator

Having a baby stimulates self-development. For the surveyed women, the transition from a non-mother woman to a mother-woman has had a formative dimension—it has been a transformation from a child into an adult.

*…when my firstborn appeared, when my daughter was born. It was the most life-changing for me because I had to change away from a little girl that didn’t worry about anything, to **become an adult**, to fret over another person, for a child (…) **I matured** right away; I immediately became an adult. Just as I was a carefree person who didn’t worry about anything, just like that, I turned into a lioness. I started fighting for my family for everything.*

*(woman, 40 yo [52])*

It is likely that there is a link between the meaning derived from being a mother and a mother’s ability to rebuild her personal and maternal identity. Identification with being a mother stimulates personal growth as women become responsible for meeting their child’s needs and excel in doing so, as well as changing their own perception of themselves in relation to others, especially the child [41]. Referring to normative concepts of development, the transition experienced by women from childhood to adulthood through the fact of taking on the responsibilities of being a mother is the realization of the classical view of human development according to the current life-span psychology. According to R. Havighurst’s developmental task theory, taking on parental tasks is a developmental task for early adulthood (between the ages of 20 and 35) and is, according to this view, a condition for further development [42]. In turn, according to the psychosocial concept of E. Erikson [43,44], the effect of overcoming the sixth developmental crisis concerning the conflict between intimacy and isolation is to build a lasting relationship with a partner and start a family (including having a child). The realization of another developmentally beneficial pole, according to Erikson’s views, means generativity, which in turn protects the individual from stagnation and arrested development. In this sense, motherhood is one of the important stimulators of development in adulthood, providing women with new challenges, experiences, skills, and competencies. The results of our study show that women are aware of the momentous role of this experience, and it is certainly a valuable personal resource, as well as a kind of protective factor against developmental stagnation.

In the later stages of adulthood, based on the process of overcoming person–context transaction conflicts [45] we assume that the meaning drawn from being a mother leads to further maturation, self-integration, and wisdom.

## 4. Discussion

The notion of transition to motherhood has been studied predominantly in the short-term spans [14,25]. The core of the analysis is the process of women adapting to the event of the first childbirth. Meanwhile, these events hold long-term significance and for many decades remain pivotal in women’s self-assessment of their lives, as demonstrated by the results of female cases in middle and late adulthood presented in the article. Transition to motherhood leaves a permanent mark in the biographical memory of women. It is reconstructed as life-changing regardless of the time that elapsed from its initiation.

The research question was whether and to what extent the meaning of the transition to motherhood changes for women from different birth cohorts.

The conducted analyses made it possible to conclude that regardless of the passage of time and the biographical perspective constituting the background of the studied narratives, giving birth to a child (especially the first child) constitutes a turning point in women’s lives. For the majority of the women surveyed, the transition was made, in line with the model by Leonia Sugarman [32], by bestowing a personal meaning to a turning point and including it in the history of one’s own life (also in the sense of identity—being a woman-mother). As shown by the analysis of the narratives by women in their middle and late adulthood, the passage of time blurs in their memory the markers typical for this event, such as information about the pregnancy, its course or the course of the delivery. The perspective of time, extended for the entire duration of a woman’s life, reveals the importance of the transition to motherhood as a constituent of the continuity, integrity and coherence of the sense of identity in women in their 40s, 50s, and 60s. One can state, following the anthropologist Arnold von Gennep [46], that a woman who becomes a mother acquires a new status when she gives birth to a child. Yet, the complete adoption of this status is a process that is subject to further reconstruction.

The conducted analyses enabled establishing that the category of change in the transition to motherhood is a standard feature for all surveyed women—in middle as well as late adulthood. In both older generations, some women remembered their first child’s birth as a coping with biographical change. From their narratives, criticality indicators were distinguished, such as potential bivalence, the unpredictability of effects, or long-term changes in various areas of their lives. The thought that seems particularly impressive and noteworthy is that the generation of women in late adulthood indicates definitely fewer difficulties related to their own motherhood. The semantic dominant of the narratives studied is motherhood as taking over responsibility for another person. Women have a powerful, unconditional, and lasting emotional bond.

For many women, the transition to motherhood is continuous. It evolves along with the changes in the field of caring and educational functions. Still, despite these changes, it remains a permanent self-identification. The transition to motherhood is an example of an endless series of changes, often recreated and integrated many times. Thus, it proves that giving birth to a child is a significant biographical life event. For young women, the experience of motherhood is strongly refocused on the physical sphere, often competing with the previously dominant role of the (sexual?) partner, providing ambivalent feelings about one’s own body (the pain of childbirth, the pleasure and/or frustration of breastfeeding the child, to name just two) [13,14]. Meanwhile, among women in middle and late adulthood, the transition to motherhood is perceived more in terms of their generativity. Becoming mothers has led directly and/or indirectly to the success of future generations—their children and their children’s children.

We are aware of no qualitative study that has clarified the lasting importance of the transition to motherhood among women in middle and late adulthood. The results of the research, although preliminary, suggest that women’s attitude towards the transition to motherhood is curvilinear. Their prospects after becoming a mother undergo a positive change in their assessment. Motherhood is a core and permanent element of their identity and self-esteem, on the basis of which they revise their value system and life goals. 

Motherhood could also be included as a resource in models of care that can be developed to facilitate women’s successful ageing. If motherhood regains its foothold over the role of grandmother, the shared behavioural expectations that arise from social interaction will increase in scope and thus may improve the well-being of ageing women. The meaning derived from motherhood may help ageing women struggling with the feeling of social transparency and diminishing self-esteem by allowing them to reconsider their views on selfness and thus enhance their existential well-being.

Increased “mother-ness” and mother confidence may also facilitate health behaviour change [47]. We are aware of the need for further exploration of this topic. A review of the literature shows inconsistent evidence that female multiparity is associated with an increased risk of maternal mental health problems [48,49]. It is possible that in the later decades of their lives, the multiplied experience of being a mother increases the ability to mobilize resources to support their ageing and provides a more favourable context for the reconstruction of the female self. This self-growth process indicates newer scope than personality or genes to develop ways to improve subjective well-being [50]. 

## 5. Conclusions

Transition, as completed, assessed by women as beneficial from the perspective of the end of their lives, constitutes a permanent identity change. Women always see themselves as women-mothers. This is significant because an ageing woman is habitually described as a grandmother than a mother in the literature on the subject. Meanwhile, as Astrid Tokaj and Danuta Krzysztofiak put it:

*every older woman who is a mother has her own unique history of motherhood. Looking from the old-age perspective, a lot of time has passed since she entered this role. It remains in the older woman’s memory until the end of her life, or until the pathological old age disturbs the possibility of free insight into past events. Undoubtedly, from her perspective (and thus from a multiyear standpoint), the reflection on the course of motherhood—its faces, conditions, experiences constituting it, and finally, the character continued in old age—has significant value [31].*

(p. 202)

The purpose of our study was primarily to examine how women in middle and late adulthood perceive their transition to motherhood. We examined if time perspective changes the meaning attributed to it. In the completed study, we received a reconstruction of life-changing events in the biographies of the surveyed women and, consequently, the conclusion that in the self-narratives of women in middle and late adulthood, the dominant identity transition in adulthood is the transition to motherhood. This significant research conclusion prompts the need for in-depth research in the discussed area. Much would be contributed by a project carried out in a longitudinal approach. Then the value of the transition to motherhood could be treated as a mediator in the process of the reconstruction of the identity (body image, sexuality and social roles) of women in middle and late adulthood. A comparative research project is planned to follow, where (in addition to the area of soma, polis, and psyche) the cultural change of expectations, patterns, and values towards women in middle and late adulthood will be studied. This could also be a valuable observation in the context of the dynamics of the family crisis raised in social discourse.

Our measurements did not escape the limitations resulting mainly from the adopted qualitative research methodology. Participants were allowed to self-express freely which resulted in some cases of narrative superficiality and made it impossible to obtain data on coping in the sample of women in middle and late adulthood. At the same time, we lack other relevant information about women (e.g., how many children they had and at what age they first became mothers) to better understand attitudes toward motherhood in middle and late adulthood. In addition, the study did not take into account the dynamics in subjective perceptions of motherhood over time, an issue that would require the use of a longitudinal study. We are aware of the need for further exploration of this topic.

## Figures and Tables

**Table 1:** Characteristics of research trials.

		Birth Cohorts	
		Middle Adulthood	Late Adulthood
Sample Size (N)		50	52
Age	Min.	40	60
	Max.	59	84
	M	47.66	69.35
	SD	5.15	6.89
Marital status	Single (N)	1	1
	Married (N)	41	30
	Divorced (N)	8	5
	Widowed (N)	-	16
Education	Primary (N)	0	9
	Vocational (N)	8	13
	Secondary (N)	17	17
	Tertiary (N)	25	13

Source: data from own research.

## Data Availability

The data presented in this study are available on request from the corresponding author. The data are not publicly available due to privacy restrictions.
